# Nuclear export as a therapeutic vulnerability in ZFTA-RELA ependymoma

**DOI:** 10.1093/neuonc/noag100

**Published:** 2026-05-03

**Authors:** Siri M Ippagunta, Amir Arabzade, Srinidhi Varadharajan, Erik Emanus, Amanda Bland, Alisha Kardian, Nicholas Laboe, Hua Sun, Tuyu Zheng, Amelia Hancock, Mark Zhai, Emma Caesar, Maxwell Park, Wilda Orisme, Melissa Johnson, Brittany Rodgers, Gregory H Turner, Jeffrey Steinberg, Burgess Freeman, Martine F Roussel, Anang Shelat, Richard W Kriwacki, Thomas E Merchant, Benjamin Deneen, Michael D Taylor, Sameer Agnihotri, Shondra Pruett-Miller, Jacob A Steele, Anoop Murthy Kavirayani, David W Ellison, Arzu Onar-Thomas, Giles W Robinson, Stephen C Mack, Kelsey C Bertrand

**Affiliations:** Department of Developmental Neurobiology, St. Jude Children’s Research Hospital, Memphis, Tennessee, USA; Neurobiology and Brain Tumor Program, St. Jude Children’s Research Hospital, Memphis, Tennessee, USA; Center of Excellence in Neuro-Oncology Sciences, St. Jude Children’s Research Hospital, Memphis, Tennessee, USA; Department of Developmental Neurobiology, St. Jude Children’s Research Hospital, Memphis, Tennessee, USA; Neurobiology and Brain Tumor Program, St. Jude Children’s Research Hospital, Memphis, Tennessee, USA; Center of Excellence in Neuro-Oncology Sciences, St. Jude Children’s Research Hospital, Memphis, Tennessee, USA; Department of Chemical Engineering, Rice University, Houston, Texas, USA; Department of Developmental Neurobiology, St. Jude Children’s Research Hospital, Memphis, Tennessee, USA; Neurobiology and Brain Tumor Program, St. Jude Children’s Research Hospital, Memphis, Tennessee, USA; Center of Excellence in Neuro-Oncology Sciences, St. Jude Children’s Research Hospital, Memphis, Tennessee, USA; Department of Developmental Neurobiology, St. Jude Children’s Research Hospital, Memphis, Tennessee, USA; Neurobiology and Brain Tumor Program, St. Jude Children’s Research Hospital, Memphis, Tennessee, USA; Center of Excellence in Neuro-Oncology Sciences, St. Jude Children’s Research Hospital, Memphis, Tennessee, USA; Department of Developmental Neurobiology, St. Jude Children’s Research Hospital, Memphis, Tennessee, USA; Neurobiology and Brain Tumor Program, St. Jude Children’s Research Hospital, Memphis, Tennessee, USA; Center of Excellence in Neuro-Oncology Sciences, St. Jude Children’s Research Hospital, Memphis, Tennessee, USA; Department of Developmental Neurobiology, St. Jude Children’s Research Hospital, Memphis, Tennessee, USA; Neurobiology and Brain Tumor Program, St. Jude Children’s Research Hospital, Memphis, Tennessee, USA; Center of Excellence in Neuro-Oncology Sciences, St. Jude Children’s Research Hospital, Memphis, Tennessee, USA; Cancer and Cell Biology Program, Baylor College of Medicine, Houston, Texas, USA; Department of Developmental Neurobiology, St. Jude Children’s Research Hospital, Memphis, Tennessee, USA; Neurobiology and Brain Tumor Program, St. Jude Children’s Research Hospital, Memphis, Tennessee, USA; Center of Excellence in Neuro-Oncology Sciences, St. Jude Children’s Research Hospital, Memphis, Tennessee, USA; Department of Developmental Neurobiology, St. Jude Children’s Research Hospital, Memphis, Tennessee, USA; Neurobiology and Brain Tumor Program, St. Jude Children’s Research Hospital, Memphis, Tennessee, USA; Center of Excellence in Neuro-Oncology Sciences, St. Jude Children’s Research Hospital, Memphis, Tennessee, USA; Department of Developmental Neurobiology, St. Jude Children’s Research Hospital, Memphis, Tennessee, USA; Neurobiology and Brain Tumor Program, St. Jude Children’s Research Hospital, Memphis, Tennessee, USA; Center of Excellence in Neuro-Oncology Sciences, St. Jude Children’s Research Hospital, Memphis, Tennessee, USA; Department of Developmental Neurobiology, St. Jude Children’s Research Hospital, Memphis, Tennessee, USA; Neurobiology and Brain Tumor Program, St. Jude Children’s Research Hospital, Memphis, Tennessee, USA; Center of Excellence in Neuro-Oncology Sciences, St. Jude Children’s Research Hospital, Memphis, Tennessee, USA; Department of Developmental Neurobiology, St. Jude Children’s Research Hospital, Memphis, Tennessee, USA; Neurobiology and Brain Tumor Program, St. Jude Children’s Research Hospital, Memphis, Tennessee, USA; Center of Excellence in Neuro-Oncology Sciences, St. Jude Children’s Research Hospital, Memphis, Tennessee, USA; Department of Developmental Neurobiology, St. Jude Children’s Research Hospital, Memphis, Tennessee, USA; Neurobiology and Brain Tumor Program, St. Jude Children’s Research Hospital, Memphis, Tennessee, USA; Center of Excellence in Neuro-Oncology Sciences, St. Jude Children’s Research Hospital, Memphis, Tennessee, USA; Department of Developmental Neurobiology, St. Jude Children’s Research Hospital, Memphis, Tennessee, USA; Neurobiology and Brain Tumor Program, St. Jude Children’s Research Hospital, Memphis, Tennessee, USA; Center of Excellence in Neuro-Oncology Sciences, St. Jude Children’s Research Hospital, Memphis, Tennessee, USA; Department of Developmental Neurobiology, St. Jude Children’s Research Hospital, Memphis, Tennessee, USA; Center for In Vivo Imaging and Therapeutics, St. Jude Children’s Research Hospital, Memphis, Tennessee, USA; Center for In Vivo Imaging and Therapeutics, St. Jude Children’s Research Hospital, Memphis, Tennessee, USA; Center for In Vivo Imaging and Therapeutics, St. Jude Children’s Research Hospital, Memphis, Tennessee, USA; Center for In Vivo Imaging and Therapeutics, St. Jude Children’s Research Hospital, Memphis, Tennessee, USA; Neurobiology and Brain Tumor Program, St. Jude Children’s Research Hospital, Memphis, Tennessee, USA; Department of Tumor Cell Biology, St. Jude Children’s Research Hospital, Memphis, Tennessee, USA; Department of Chemical Biology and Therapeutics, St. Jude Children’s Research Hospital, Memphis, Tennessee, USA; Department of Structural Biology, St Jude Children's Research Hospital, Memphis, Tennessee, USA; Cancer Biology Program, St. Jude Children’s Research Hospital, Memphis, Tennessee, USA; Department of Radiation Oncology, St. Jude Children’s Research Hospital, Memphis, Tennessee, USA; Center for Cancer Neuroscience, Baylor College of Medicine, Houston, Texas, USA; Texas Children’s Hospital, Baylor College of Medicine, Houston, Texas, USA; Department of Neurological Surgery, University of Pittsburgh, Pittsburgh, Pennsylvania, USA; Center for Advanced Genome Engineering, St. Jude Children’s Research Hospital, Memphis, Tennessee, USA; Center for Advanced Genome Engineering, St. Jude Children’s Research Hospital, Memphis, Tennessee, USA; Department of Pathology, St. Jude Children’s Research Hospital, Memphis, Tennessee, USA; Neurobiology and Brain Tumor Program, St. Jude Children’s Research Hospital, Memphis, Tennessee, USA; Department of Pathology, St. Jude Children’s Research Hospital, Memphis, Tennessee, USA; Neurobiology and Brain Tumor Program, St. Jude Children’s Research Hospital, Memphis, Tennessee, USA; Department of Biostatistics, St. Jude Children’s Research Hospital, Memphis, Tennessee, USA; Neurobiology and Brain Tumor Program, St. Jude Children’s Research Hospital, Memphis, Tennessee, USA; Department of Oncology, St Jude Children's Research Hospital , Memphis, Tennessee, USA; Department of Developmental Neurobiology, St. Jude Children’s Research Hospital, Memphis, Tennessee, USA; Neurobiology and Brain Tumor Program, St. Jude Children’s Research Hospital, Memphis, Tennessee, USA; Center of Excellence in Neuro-Oncology Sciences, St. Jude Children’s Research Hospital, Memphis, Tennessee, USA; Neurobiology and Brain Tumor Program, St. Jude Children’s Research Hospital, Memphis, Tennessee, USA; Department of Oncology, St Jude Children's Research Hospital , Memphis, Tennessee, USA

**Keywords:** ependymoma, therapeutics

## Abstract

**Background:**

Ependymoma (EPN) is the third most common pediatric brain tumor with no targeted therapies available to patients. In supratentorial ependymoma, the most frequent driver alteration is a gene fusion between *ZFTA* and *RELA* (denoted ZR), which leads to constitutive localization of ZR in the nucleus. Because ZR is not currently druggable, we tested whether ZR expression leads to aberrant protein interactions that could represent therapeutic vulnerabilities.

**Methods:**

Using CRISPR-Cas9 pooled screening, we identified many novel druggable ZR-interacting proteins including XPO1, CARM1, SMARCA4, and CDK1. We focused on the nuclear export protein (XPO1), given the ability of most XPO1 inhibitors (ie, selinexor) to cross the blood-brain barrier, FDA approval, and have documented safety profiles in children.

**Results:**

We found that specific nuclear ZR levels are needed for cell proliferation and are regulated by XPO1. Increased ZR accumulation in the nucleus does not increase oncogenic gene expression but drives tumor cells out of the cell cycle, as compared to a defective ZR DNA-binding mutant. Treatment of ZR-driven patient-derived mouse models with selinexor impairs cell growth and extends survival of animals *in vivo*. The combination of selinexor treatment with gemcitabine and ribociclib (used in a clinical trial for relapsed EPN at St. Jude Children’s Research Hospital [SJDAWN]) further extends mouse survival.

**Conclusion:**

Our findings demonstrate that ZR-interacting proteins constitute therapeutic leads and that XPO1 is critical for titrating “goldilocks” levels of ZR nuclear expression. We identify a novel combination therapy of selinexor, gemcitabine, and ribociclib that may be immediately translated into clinical trials for EPN patients that are currently without targeted treatments.

Key Points

- ZFTA-RELA interactome contains cellular growth dependencies.
- Nuclear export titrates oncoprotein expression to maintain cell proliferation.
- Selinexor therapy is an effective strategy against ZFTA-RELA ependymoma.

Importance of the Study

Treatment for ependymoma (EPN) has remained largely unchanged in the last 2 decades, consisting of surgery and radiotherapy. Progress against this disease has been hampered by a lack of understanding of EPN biology, leading to few clinical trials that translate laboratory findings. Our study points to a potentially effective strategy against EPN, particularly ZFTA gene fusion-driven tumors, through inhibition of nuclear transport with brain penetrant drugs, such as selinexor. Single agent and combination therapies revealed in our study could be immediately translated to clinical trials for children and adult patients with EPN.

Pediatric ependymoma (EPN) is a malignant tumor that can be found throughout the central nervous system (CNS).^1^ Despite histologic similarities, EPN tumors are highly diverse, as DNA methylation profiling categorizes these tumors into distinct disease subgroups.^2–10^ Maximal safe surgical resection and radiation therapy remain the standard of care for EPN, while chemotherapy has shown little efficacy.^1^ Although the 5-year overall survival rate is around 70%, the 10-year survival is closer to 50%, owing to the relentless nature of this tumor leading to frequent patient relapse.^1^ Over two-thirds of supratentorial ependymomas harbor gene fusions between the Zinc-Finger Translocation Associated (*ZFTA*) gene and another gene partner, commonly a transcription factor or co-activator protein.^11^ The most common of these ZFTA-fusion oncoproteins (FOs) involves RELA (denoted ZR), one of the main components of the NF-ĸB inflammatory pathway.^11^ ZR expression alone is sufficient to drive tumorigenesis in mice through constitutive nuclear localization and aberrant DNA binding.^11–14^ This suggests that ZR represents a lead target against EPN. However, targeting ZR is challenging for several reasons: (1) ZR localization bypasses cytosolic RELA inhibition, rendering NF-ĸB inhibitors likely ineffective, and (2) ZR protein is relatively disordered, thereby hindering efforts to characterize the structure for drug discovery.^11^ In this study, we hypothesized that mis-localization of the ZR protein would lead to aberrant protein-protein interactions critical for tumor cell growth and reveal therapeutic targets. Furthermore, we tested the concept that perturbing ZR localization and nuclear function could represent a potential EPN cell vulnerability and have broader applicability to other FOs.

## Methods

### Animal and Biological Research

All studies in this article were performed with approval from the Animal Care and Use Committee (IACUC) and Institutional Biosafety Committee in accordance with best practices outlined by the NIH Office of Laboratory Animal Welfare. Mice were housed in an AAALAC-accredited facility. All mice were housed in a temperature-controlled animal room (25°C) with a 12:12-hour light:dark cycle. Mice were housed 5 per cage and fed an AIN-93G diet. All preclinical studies were performed on NSG mice (obtained from St. Jude Animal Colony). All *in utero* electroporations (IUEs) were performed in CD-1 mice (obtained from Charles River Inc.). CD-1 litters consisted of both female and male mice. The endpoint was determined by neurological symptoms or tumor burden. At the endpoint, the mice were euthanized under isoflurane anesthesia and perfused with either 10 mL PBS for freezing tumor for RNA sequencing analysis or 10 mL PFA for IHC analysis.

### Cell Culture

The HEK293T line was grown on tissue culture-treated plates using DMEM media supplemented with FBS, sodium pyruvate, glutamine, and Primocin. Mouse ZFTA-RELA ependymoma IUE models and patient-derived xenograft cell lines were grown on Matrigel-coated plates using neural basal medium (Invitrogen) supplemented with sodium pyruvate, glutamine, B27, N2, rhFGF (10 ng/mL), and rhEGF (20 ng/mL). All lines tested negative for Mycoplasma contamination, as assessed using a PCR-based approach. Tumor-derived cell lines were confirmed to be authentic and unique by short tandem repeat fingerprinting.

### Sucrose Gradient Centrifugation

Cells expressing the protein of interest are scraped off the plate and washed with ice-cold PBS once and then with 8% (w/v) sucrose solution in 0.1× PBS. Cells were then resuspended in 1.4 mL of 8% sucrose in 0.1× PBS solution, followed by the addition of 5.6 mL lysis buffer (1 mM HEPES, pH 7.2, 0.5% NP-40, 0.5mM MgCl_2,_ and 0.1% β-mercaptoethanol supplemented with 1× protease inhibitor cocktail at the time of experiment). Cells are incubated for 25-30 minutes, with agitation every 5 minutes. Solution is then centrifuged at 3000*g* for 10 minutes at 4°C. The supernatant is then filtered through a 40-mm nylon mesh. Filtered supernatant is then laid on top of a sucrose gradient solution (Gradient buffer: 10 mM PIPES pH 7.2, 1 mM EDTA, 0.1% Triton X-100, and 0.1% β-mercaptoethanol) as follows: 1.2 mL 70% (w/w), 2 mL 50% (w/w), and 2.5 mL 40% (w/w). The column is then centrifuged in a swinging bucket centrifuge at 30K rpm for 3 hours without any breaks. At the end, the bottom of the tube is pierced, and samples are collected for further analysis by Western blotting.

### Western Blot

Western blot analysis was performed using standard techniques. Briefly, cells were lysed using 1X RIPA buffer supplemented with protease inhibitor cocktail. After protein quantification using Bradford assay (for sucrose fractions, Qubit protein assay was used to quantify the protein concentration), equal amounts of protein samples were loaded on a denaturing SDS-PAGE protein gel in Tris-Glycine-SDS running buffer. Proteins were then transferred overnight at 4°C using Tris-Glycine plus 20% methanol transfer buffer. Antibodies used to probe the blots were: anti-HA antibody (Abcam 9110), b-actin (Millipore A1978), and anti-RELA antibody (CST 8242).

### CRISPR Knock-out Line Generation

sgRNA targeting RELA was designed using the CRISPR tool and cloned into a lenti-CRISPRv2 backbone using the GECKO protocol. Mouse ependymoma IUE model cells were seeded 24 hours prior to infection and were infected with freshly collected lentivirus particles using 8ug/mL polybrene for 24 hours. Knock-out efficiency was tested with Western blot on infected cells after puromycin selection.

### Immunoprecipitation and Mass Spectrometry

Mouse IUE tumor cells and CRISPR knock-out lines were grown on Matrigel-coated plates and scraped off the plate when ready. Cells were washed with ice-cold PBS and lysed with 1× RIPA buffer supplemented with 1× protease inhibitor cocktail. Protein A/G Dynabeads were washed with ice-cold PBS and incubated with anti-HA antibody for 4 hours on a rotator. After 4 hours, the beads were washed with ice-cold PBS 3 times to remove unbound antibody. Cell Lysate was then added to the beads and incubated overnight on a rotator at 4°C. Afterwards, the incubation beads were washed 3 times with ice-cold PBS to remove unbound proteins. Proteins were eluted from the beads and run on an SDS-PAGE gel. Bands were then cut from the gel, destained and trypsin digested, and finally peptides were extracted for mass spectrometry analysis.

### CRISPR/Cas9 Dependency Library Screen

A Single-stranded oligo pool that contains the gRNA library was synthesized by TWIST Biosciences. The pool of oligos was then cloned into the Lenti-CRISPR v2 backbone, and the final isolated plasmids were sequenced to ensure the equal representation of all guide RNAs in the pool. Lentivirus was then prepared using standard methods. Briefly, 293T HEK cells were transfected with the library plasmid along with helper plasmid, pMD2.G and psPAX2. Cells and the media were collected 2 days post-transfection, snap frozen to break the cells apart, and the virus was concentrated using Takara Bio Inc. Lenti-X Concentrator. To ensure that during the library screen each cell will receive 1 guide RNA, the amount of virus used during the infection was optimized so that the multiplicity of infection would fall somewhere between 0.3 and 0.4. For the dependency screen, cells were seeded on the plate the day before infection. The starting number of cells was calculated based on the desired coverage. Cells were infected with the optimized amount of virus and selected with puromycin 2 days after infection. Upon completion of selection, enough cells were frozen down that would represent the library with the desired coverage, and the same number of cells was grown for 3 weeks. Each week, similar numbers of cells were collected for genomic DNA extraction. Genomic DNA was extracted using the Qiagen DNeasy Blood & Tissue kit. The region containing the guide RNA sequence was then amplified from the genomic DNA, barcoded and then sequenced. Sequencing results were then analyzed using the MAGeCK pipeline.

### In Vitro Cell Electroporation

Electroporation was done using Mouse Neural Stem Cell Nucleofector kit following the manufacturer’s recommendations. Briefly, 1425 patient-derived xenograft (PDX) model cells were seeded 2 days prior to electroporation at roughly 40% confluency. On the day of electroporation, cells were treated with 10 mM ROCK Inhibitor for 1 hour, and 1.5 mg of endotoxin-free plasmid was used in each electroporation. Cells were supplemented with 1× CloneR2 post-electroporation to boost viability. The media was changed a few hours after electroporation to remove CloneR2. Cells were used 24 hours after electroporation for imaging or cell proliferation assays.

### In Utero Electroporation

All animal procedures in this study were performed with St. Jude Institutional Animal Care and Use Committee approval. IUE was performed as described previously,^12^ and plasmids were prepped with the NucleoBond Xtra Maxi Plus EF kit (Catalog# 740426, Takara Biosciences). After anesthesia with 4.5% isoflurane, pregnant mice at E16.5 were subjected to abdominal incision to expose the uterus. DNA plasmid cocktail (1 µg/µL pBCAG-HA-ZR^FUS^1, 1 μg/μL pbCAG-eGFP-Luciferase, 1.5 µg/µL pX330-sg*Tp53*, 2 μg/μL GLAST-PBase, 1.5 µg/µL, FastGreen dye) was injected into the lateral ventricles with a glass pipette. Electric pulses were then delivered to the embryos by gently clasping their heads with forceps-shaped electrodes. Six 33 V pulses of 55 ms were applied at 100-ms intervals. The uterus was then repositioned into the abdominal cavity, the abdominal wall was sutured, and the skin was stapled. Following birth, pups were monitored for clinical signs of tumor growth (seizures, circling, head doming, etc.) as well as by MRI every 2 weeks. In accordance with our St. Jude IACUC protocol, endpoint was defined based on a set of neurological symptoms (gait, hunching, kyphosis, squinting) and these limits were not exceeded in any experiments.

### Cell Proliferation Assay

The seeding number of 1425-NS cells within each well of a 96-well plate was first optimized by seeding cells with various densities and performing a cell titer glow assay each day for a week. Cell titer glow assays were performed by adding room temperature cell titer glow reagent (Promega G9241) to each well, with a 1:1 ratio to the media, and incubating at room temperature for 10 minutes, protected from light with shaking. The luminescence is then measured using a plate reader. The optimal seeding density had low variability among replicates, and cells remained proliferative (linear increase in luminescence value over days).

1425-NS Cells were electroporated as previously indicated. The day after electroporation, the optimized number of GFP-positive cells was sorted into each well of a 96-well plate, 4 replicates. The proliferation of cells was tracked over time using cell titer glow assay as described.

### Immunofluorescence Staining

Mouse IUE model cells were plated on Matrigel-coated number 1.5 coverslips. After 24 hours, cells were washed with PBS and fixed with 4% paraformaldehyde for 10 minutes at room temperature. Cells were then washed with PBS, 3 times for 5 minutes each time. Cells were then blocked with blocking buffer (3% goat serum, 0.1% tween-20, and 0.3M glycine in PBS) for an hour at room temperature. Coverslips were then incubated with primary antibodies diluted in blocking buffer (anti-HA tag, abcam9110) in a humidified chamber overnight at 4°. Cells were washed with PBST (PBS buffer containing 0.1% tween20) 3 times, 5 minutes each time. Coverslips were then incubated with secondary antibodies of goat origin for an hour at room temperature in a humidified chamber. After an hour, cells were washed with PBST 3 times, for 5 minutes each time. DAPI was then used to stain the nucleus. After a quick rinse with PBST to remove excess DAPI, coverslips were gently dipped in distilled water to remove residual salts, air dried slightly and were then mounted on a slide using diamond prolong mounting media. Slides were kept at room temperature and protected from light for at least 48 hours before imaging.

### Animal Cortex Implantation

On the day of injection, 1425-NS cells were taken off the Matrigel plates using Accutase. Cells were counted, and 1 million cells per injection were spun down and resuspended in a solution of 1:1 PBS and Matrigel and kept on ice. Resuspension volume was set at 5 µL per injection. Mice were anesthetized with a Ketamine/Xylazine cocktail (100 and 10 mg/kg, respectively). Following the loss of pedal reflex, scalp hair was removed, and the surgical area was aseptically prepped. Anesthetized animals were then placed on stereotaxic equipment, following which the scalp and dura mater were removed to open a cortical window, exposing the surface of the brain. Mice were then injected with cells, using 5µL of cell suspension per injection. Stereotaxic coordinates: starting at the intersection point of the sagittal sinus and bregma: X-Anterior-Posterior 2 mm, Y-Medio-Lateral 1mm, Z-Dorso-Ventral 2.0-2.5 mm. After implantation, the wound was sealed with wound clips.

### In Vivo Drug Administration

Selinexor (3×/wk) and ribociclib (7×/wk) administration was via oral gavage. Briefly, a feeding tube of appropriate size was selected based upon the consistency of the liquid and animal’s size. The tube is then placed on a syringe carefully. Substrate is drawn up while making sure to remove all air from the tube. The tube itself is then wiped to ensure no droplets are left on the outside of the tube. Using the non-dominant hand, the loose skin along the neck and back of the mouse is grasped, and the tail is tucked in under one of the fingers to provide additional stability for the animal. Using the dominant hand, the tube is directed toward the back of the throat and immediately straightened to be directly in line with the animal’s body. A gentle resistance on the tube means the tube has reached the stomach. Once the correct dose is administered, the animal is placed back in the cage and observed for any adverse reactions. Gemcitabine administration (1×/wk) was done by tail vein injection.

### Histopathology and Immunohistochemistry

Brains were dissected and fixed in 10% Neutral Buffered Formalin. Fixed brains with PDX tumors were processed using the HistoCore PEGASUS Tissue Processor (Leica Biosystems, Deer Park, IL). Processed tissues were embedded in paraffin and sectioned at a thickness of 4 µm using a HistoCore AUTOCUT fully automated rotary microtome (Leica Biosystems, Deer Park, IL). Tumor sections were stained with hematoxylin and eosin (H&E) using the HistoCore SPECTRA ST Stainer (Leica Biosystems, Deer Park, IL). Simplex immunohistochemistry (IHC) was performed to detect cleaved-caspase 3, CRM1 (XPO1), Ki67, p16^INK4A^, p21, p53, p62 (SQSTM1), p65 (RELA), and pH2AX (gamma H2AX). H&E and IHC slides were coverslipped using the HistoCore SPECTRA CV Coverslipper (Leica Biosystems, Deer Park, IL). Pertinent IHC details are summarized in Table S5.

IHC protocols to detect the aforementioned markers were performed on one of the following automated immunostainers:

- Leica Bond Rx (Leica Biosystems, Deer Park, IL).
- Ventana BENCHMARK ULTRA (Roche Diagnostics Corporation, Indianapolis, IN).
- Ventana Discovery Ultra (Roche Diagnostics Corporation, Indianapolis, IN).

H&E and IHC slides were reviewed by a board certified veterinary anatomic pathologist (A.M.K.) using an Olympus BX46 microscope (Evident Scientific, Waltham, MA) with additional input from a senior medical neuropathologist (D.W.E.). Representative light microscopic images were acquired using a Spot Insight Color Mosaic microscope camera (Spot Imaging, Sterling Heights, MI). Whole slide images with an effective scalable magnification of 32× were generated using the 20× objective of a PANNORAMIC 250 Flash III digital slide scanner (3DHISTECH Ltd, Budapest, Hungary). Quantitative analysis of selected immunohistochemistry markers was performed using the HALO (v3.6.4134.137) digital pathology software program (Indica Labs, Albuquerque, NM).

### Magnetic Resonance Imaging

MRI was performed on a Bruker Biospec 94/20 MRI system (Bruker Biospin MRI GmbH, Ettlingen, Germany). Prior to scanning, mice were anesthetized in a chamber (3% isoflurane in oxygen delivered at 0.5 L/min) and maintained using nose-cone delivery (1-2% Isoflurane in oxygen delivered at 0.2 L/min). Animals were provided thermal support using a heated bed with warm water circulation and a physiological monitoring system to monitor breath rate. MRI was acquired with a mouse brain cryoprobe positioned over the mouse head and placed inside an 86-mm transmit/receive coil. After the localizer, T2-weighted Rapid Acquisition with Refocused Echoes sequences were performed in the coronal (TR/TE = 2000/20.4 ms, matrix size = 256 × 256, field of view = 20 mm × 20 mm, slice thickness = 0.5 mm, number of slices = 16) and axial (TR/TE = 2500/23 ms, matrix size = 256 × 256, field of view = 20 mm × 20 mm, slice thickness = 0.5 mm, number of slices = 32) orientations. Tumor volumes were analyzed by using 3D Slicer software.


$$\mathrm{Tumor}\;\mathrm{volume}\;\%\;\mathrm{change}\;=\frac{\left((\mathrm{Final}\;\mathrm{tumor}\;\mathrm{volume}-\mathrm{Initial}\;\mathrm{tumor}\;\mathrm{volume})\cdot 100\right)}{\mathrm{Initial}\;\mathrm{tumor}\;\mathrm{volume}}$$

Initial tumor volume = tumor volume 3 days pre-treatment period

Final tumor volume = tumor volume 30 days post-treatment period

### Sequencing Data Analysis

Adapter trimming was performed on raw RNA sequencing reads using Trimgalore. The resulting reads were aligned to mm10 using STAR (v2.7.11b). Read counts were quantified with a Gencode (v27) reference annotation using htseq-count, followed by differential expression analysis using DEseq2. Lowly expressed genes, with count summed over all samples below 10, were filtered out from the input matrix to DESeq2. Pathway analysis was performed using ClusterProfiler.

## Results

### ZFTA Fragment Drives Aberrant Protein-Complex Association in Mouse and Human EPN

Given the simultaneous mis-localization of both ZFTA and RELA in the process of ZR gene fusion,^15^ we hypothesized that ZR would engage distinct sub-cellular compartments. To test this hypothesis, we utilized a sucrose gradient centrifugation method in which protein complexes are separated from each other based on their buoyant density (Figure 1A). Using this method, we subjected cellular extracts containing an HA-tagged version of ZR to a column of sucrose solutions with different concentrations and collected samples from different parts of the column to analyze by Western blot. While the RELA protein tended to fall within the lower density portions of the column, ZR had a strikingly different pattern of presence within the column, occurring at a higher density (Figure 1B). Interestingly, fusing the ZFTA fragment to monomeric cherry fluorescent (mCherry) protein had a similar change in its pattern within the column, suggesting that the ZFTA fragment was likely causing this behavior (Figure 1C). To validate this observation in the human disease, we subjected cell extracts from ZR-driven PDX models and tested the extracts with a RELA antibody that would recognize both the endogenous RELA protein as well as the ZR fusion protein. We observed a difference between the patterns for endogenous RELA protein and ZR fusion protein in patient-relevant disease models (Figure 1D and E). Similar patterns were also seen in cell lines from a genetic mouse model of ZR ependymoma generated by IUE of the developing mouse brain (Figure 1F). We then analyzed the protein contents of 3 separate parts of the column. Fraction 1 had the highest sucrose concentration but almost no ZR protein, Fraction 2, where ZR was enriched the most, and Fraction 3, where the sucrose concentration was the lowest and lacked any signal for ZR presence (Figure 1G, Table S1). Within the ZR containing Fraction 2, we observed a high enrichment of many high molecular weight protein complexes such as spliceosome complex, coated vesicles, and the pericentriolar complex. These findings suggest that the ZR protein is associated with distinct high molecular weight protein complexes of unknown functional significance, and this behavior is predominantly mediated by the ZFTA protein fragment that undergoes gene fusion in EPN.

**Figure 1.**
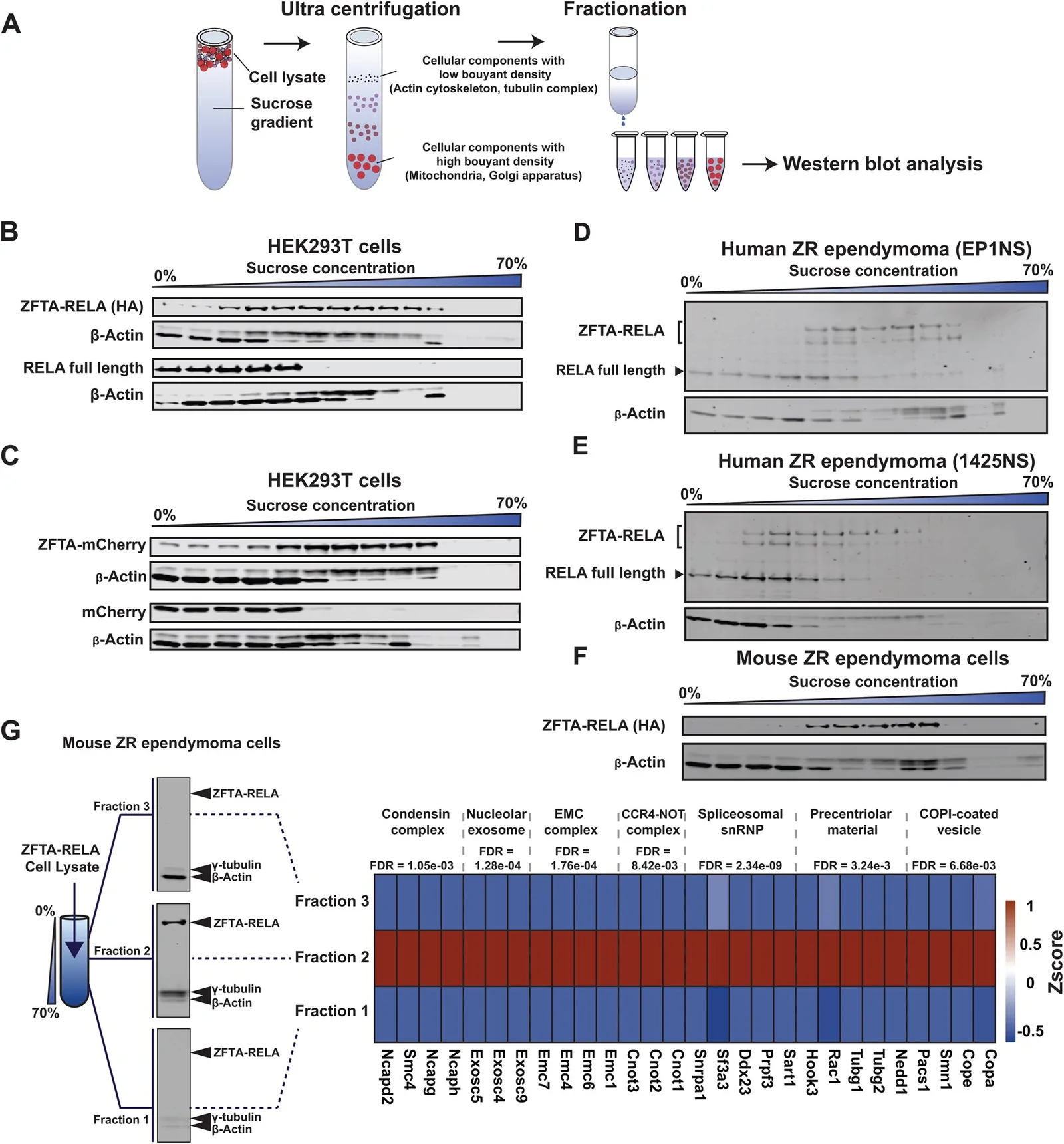
(A) Schematic representation of cellular fractionation by sucrose gradient centrifugation. Cell extracts are laid on top of a sucrose gradient and complexes are separated after ultracentrifugation based on their buoyant density. Fractions are collected and analyzed by Western blot. (B) Western blot analysis of sucrose gradient fractionation, using HEK293T cell extracts expressing either ZFTA-RELA or RELA proteins. Proteins were probed using an anti-HA tag antibody. (C) Western blot analysis of sucrose gradient fractionation, using HEK293T cell extracts expressing either mCherry-tagged ZFTA or mCherry protein alone. Proteins were probed using an anti-HA tag antibody. (D) and (E) Western blot analysis of sucrose gradient fractionation, using 2 different patient-derived xenograft models, EP1NS and 1425NS, respectively. Blots were probed using anti-RELA antibodies, which recognize both ZFTA-RELA and endogenous RELA proteins. (F) Western blot analysis of sucrose gradient fractionation, using cell extracts from a ZR ependymoma mouse IUE model. (G) Mass spec analysis of 3 different fractions from the sucrose gradient and GO analysis of some of the enriched protein complexes.

### ZFTA-RELA Protein Interactome Reveals Therapeutic Targets

We hypothesized that aberrant ZR localization would lead to new protein-protein interactions important for cell proliferation and tumor growth. While ZR protein interactions have been described in human embryonic kidney epithelial—Large T transformed (HEK293T) cells expressing ZR, such experiments have yet to be performed in native tumor models.^13^^,^^16^ To this end, we sought to identify protein-protein interacting partners of ZR in our genetic IUE mouse model. We performed immunoprecipitation followed by mass spectrometry and compared interacting proteins against a ZR knock-out control, in which ZR was targeted using CRISR-Cas9 gene editing (Figure 2A, Figure S1A and B). We observed significant enrichment of many members of the NF-ĸB pathway such as NFKB1 and NFKB2, which are consistent with interactions detected in human cells^16^ (Figure 2A). Among the top protein-protein interacting partners of ZR, we found members of RNA splicing machinery (FXR1 and NCL), MCM complex (MCM 2,5,6,7), cyclin-dependent kinases (CDK1), chromatin remodeling proteins (SMARCC2), and regulators of protein export (XPO1, RAN, HSPA9) (Figure 2A, Figure S1C and Table S2).

**Figure 2.**
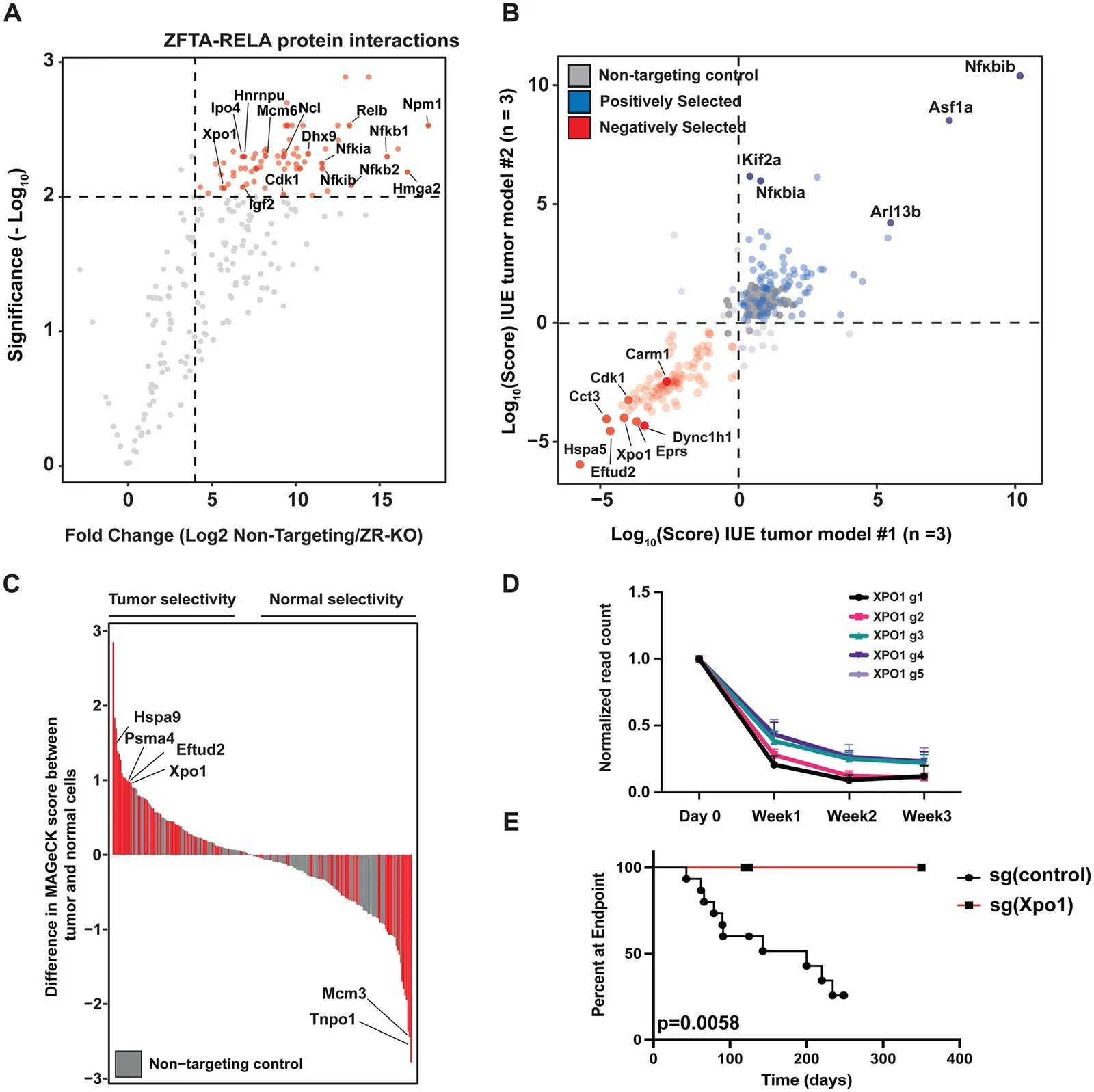
(A) Volcano plot analysis of ZFTA-RELA interacting proteins. (B) Gene scores for 2 biological replicate mouse ZR models in a CRISPR dependency screen. Genes highlighted with blue are positively selected (tumor suppressor), and genes highlighted with red are negatively selected (tumor dependency). Non-targeting guides are depicted with gray color. (C) Dependency genes ranked based on their selectivity toward tumor cells or neural stem cells. (D) Normalized read counts of sgRNAs targeting Xpo1 across 3 weeks in the EPN IUE mouse cell line model. (E) Survival of IUE ZR-driven EPN mice co-electroporated with either non-targeting sgRNA or sgRNA targeting Xpo1.

To test whether cells derived from our mouse IUE model were dependent on specific ZR-interacting partners, we developed a focused CRISPR/Cas9 pooled drop-out assay against 207 genes (5 sgRNA/gene), and 105 non-targeting sgRNA controls (Figure 2B, Table S3). The CRISPR/Cas9 drop-out screen was performed in cell cultures from 2 separate IUE mouse models, serving as biological replicates with 3 technical replicates per experiment (Figure 2B, *n* = 6). sgRNA depletions and enrichments were scored using the MAGeCK algorithm.^17^ Non-targeting guide RNAs (gray dots) all clustered near the center of the graph, indicating no selection pressure or global changes in library representation. Interestingly, we found that sgRNAs targeting inhibitors of the NF-ĸB pathway (namely *Nfkbia* and *Nfkbib*) were significantly enriched in the screen, indicating a potential tumor suppressive role of inhibitors of the NF-ĸB pathway (Figure 2B). Conversely, sgRNAs that were depleted included *Xpo1, Cdk1, Hspa5, Cct3,* and *Eftud2*, representing potential cell proliferative vulnerabilities and targets for therapy (Figure 2B). To test the tumor specificity of these “hits,” we performed the same screen in radial glial progenitor cells, which represent candidate cells of origin of EPN tumors.^18^ We then compared the scores from our ZR IUE EPN models to the scores in normal radial glial cells and ranked them by tumor selectivity (Figure 2C, Figure S1D). Among the top-ranking tumor-specific “hits” that included *Carm1*, *Hspa9*, *Nifk*, and *Xpo1*, we decided to move forward with Exportin 1 (*Xpo1*), given the availability of FDA approved and BBB penetrant drugs targeting this protein (Table S4). XPO1 plays a critical role in regulating nuclear export of a variety of cargoes including proteins and RNA molecules, including tumor suppressor proteins such as TP53.^19^ Dysregulation of this protein has been linked to a variety of malignancies and has been associated with resistance to several chemotherapy agents.^20^^,^^21^ XPO1 KO in our IUE-derived cell lines inhibits the growth and tumorigenesis in our IUE model (Figure 2D and E, Figure S1E and F). Taken together, our findings indicate that the protein interaction partners of ZR fusion protein (such as XPO1) represent important cell proliferative vulnerabilities and constitute potential therapeutic targets against the disease.

### Precise Nuclear Levels of ZR Are Required for Tumor Cell Proliferation

ZR fusion protein leads to its constitutive localization in the nucleus.^11^ However, we have found that extended expression of ZR in neural stem cells selects for cells that export excess ZR protein to the cytosol (Figure 3A). This led us to hypothesize that ZR localization is XPO1 mediated, and that “precise” levels of nuclear expression is needed for cell survival. Supporting this idea, selinexor treatment led to significant re-localization of ZR to the nucleus (Figure 3A and B). Critically, selinexor-driven ZR re-localization did not significantly upregulate ZR target genes, suggesting a maximal saturation of ZR target binding (Figure 3C, Figure S2A). Rather, ZR increase in the nucleus led to neuronal maintenance and ion channel pathway upregulation, suggesting a possible impact on cellular differentiation state (Figure S2B). To test the hypothesis that excessive ZR levels were associated with reduced cell proliferation, we utilized electroporation to express excess ZR protein in a ZR-driven ependymoma patient model. While exogenous ZR expression inhibited cell proliferation, a zinc-finger DNA-binding defective mutant, denoted ZR-ΔZF known to inhibit ZR nuclear localization continues to grow in a similar pattern as GFP-expressing control cells (Figure 3D, Figure S2C).^16^ This led us to posit that finely tuned “goldilocks” levels of ZR nuclear expression are required for continued cell proliferation, and that excessive ZR nuclear expression inhibits cell growth. Finally, to evaluate whether human ependymoma cell lines are sensitive to XPO1 inhibition, we performed cell proliferation assays on a number of PDX models driven by ZFTA-fusion proteins. All tested cell lines were sensitive to XPO1 inhibition by selinexor treatment at IC50 concentrations <150 nM (Figure 3E, Figure S2D). This is relevant since these drug concentrations are achievable in the mouse brain.^22–24^ Taken together, our findings reveal a cell proliferative vulnerability in ZR ependymoma by exacerbation of ZR nuclear localization using inhibitors that target XPO1.

**Figure 3.**
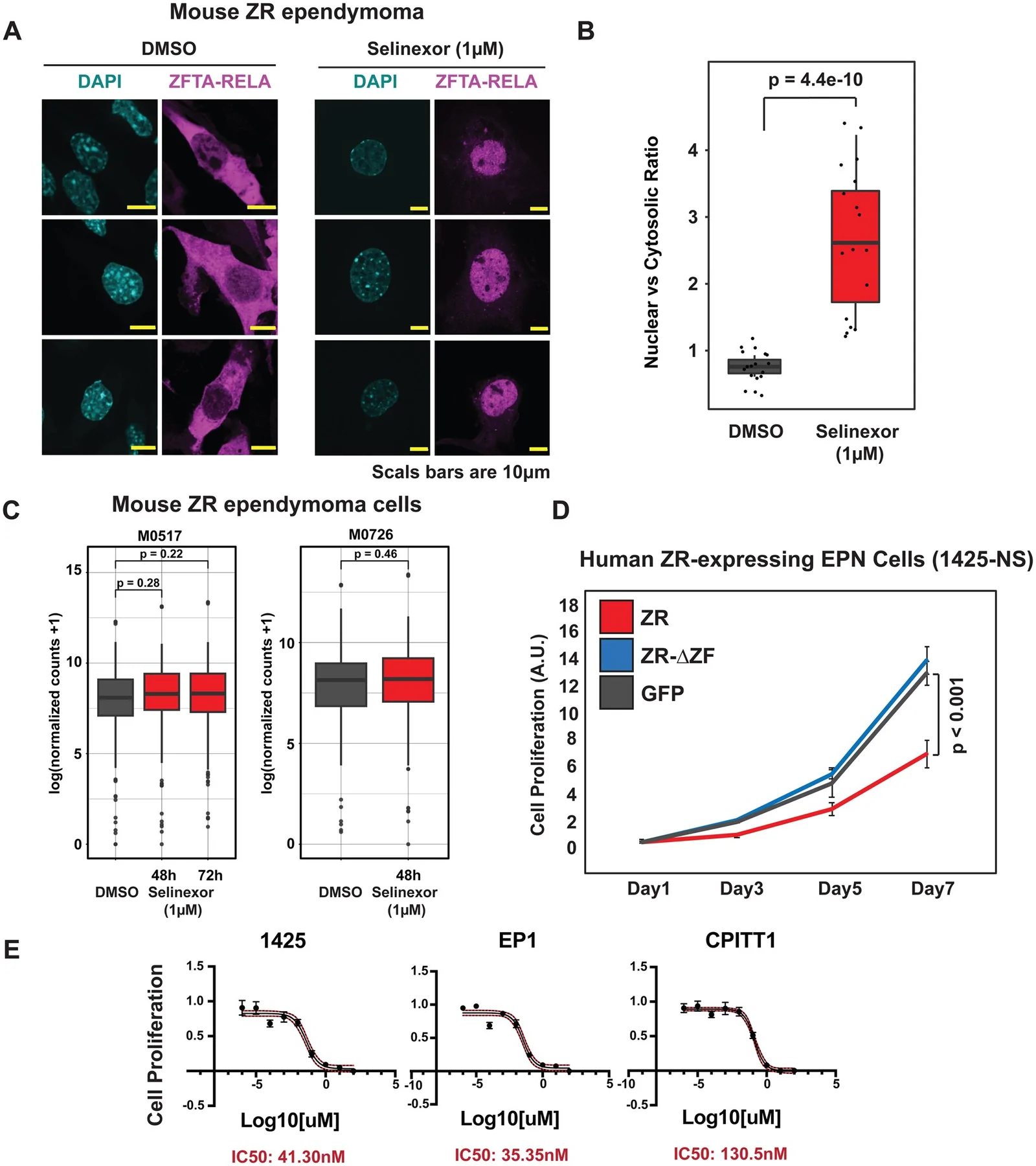
(A) Representative images of mouse ZR ependymoma cells treated with DMSO or 1µM selinexor for 24 hours. ZFTA-RELA is stained using an anti-HA tag antibody, and the nucleus is marked with DAPI. (B) Nuclear to cytoplasmic ratio of mouse ZR ependymoma cells treated with DMSO or 1 µM selinexor for 24hrs. (C) Read count comparison for ZFTA-RELA downstream target genes for 2 mouse ZR ependymoma models treated with DMSO or 1 µM selinexor for 48 or 72 hours. (D) Cell proliferation of the human ependymoma xenograft model after electroporation with each of the indicated constructs. (E) ZFTA-fusion patient-derived xenograft models treated with increasing concentration of selinexor with cell proliferation measured using an Alamar Blue assay. Red lines indicate the 95% confidence margins.

### Selinexor Combination Therapy Is Effective Against a ZR-Driven Mouse Model

Selinexor has shown promise as a potential treatment for several malignancies including refractory multiple myeloma, pancreatic,^25^ and prostate^26^ cancer, all in preclinical and early phase clinical trials.^27^^,^^28^ Further, selinexor has shown improved efficacy in combination with other targeted and chemotherapies.^26^^,^^29^ To evaluate selinexor therapy in an EPN murine model, we used a ZR-driven PDX model (named 1425-NS).^30^ Because monotherapies are unlikely to translate to meaningful survival benefits in patients, we also evaluated selinexor in combination with gemcitabine and ribociclib (GR),^31^ which have previously undergone evaluation in human EPN clinical trials at St. Jude Children’s Research Hospital. Screening of the Washington University Drug Gene interaction Database also identified gemcitabine as an inhibitor of Cdc5l, another “hit” from our CRISPR screen; Table S4).^32^ A mouse preclinical trial was performed in which tumor progression was tracked by MRI at weeks 3, 6, 8, 10, and 12, and drug dosing began at the first signs of tumor presence by MRI (Figure 4A). Mice were divided into vehicle and drug arms at random and treated with selinexor administered 3 days/wk, gemcitabine (1 day/wk), and ribociclib (7days/wk) for two 4-week cycles with a 1-week drug holiday between cycles (Figure 4A). The reason for the drug holiday was to mimic treatment strategies pursued in a human setting, and also to address the drug tolerability in mice with continued dosing. We demonstrate that in the ZR-driven 1425-NS model, selinexor monotherapy significantly extended survival and impaired tumor growth while on therapy (Figure 4B and C). These findings were potentiated by the addition of GR, which further improved survival outcomes of mice treated with selinexor, gemcitabine, and ribociclib (SGR) (Figure 4B and C, Figure S3A and B). Tissue isolated while on therapy was subjected to immunohistochemical analysis (IHC), which demonstrated increased cleaved Caspase 3 in response to selinexor therapy (Figure 4D and E). Because there are no antibodies that recognize ZR specifically, we tested whether IHC analysis of RELA might serve as a potential biomarker for nuclear accumulation of ZR. We observed no significantly detectable differences when staining for RELA, likely a result of cross-reaction against endogenous RELA nuclear protein (Figure S3C). However, we did observe significant accumulation of nuclear P62 after selinexor treatment, representing a potential biomarker to evaluate tumor cell responses to selinexor treatment (Figure 4F, Figure S3D). Together, our findings demonstrate that selinexor treatment is an effective therapy against ZR fusion-driven EPN in mice, and these effects can be further improved in combination with GR.

**Figure 4.**
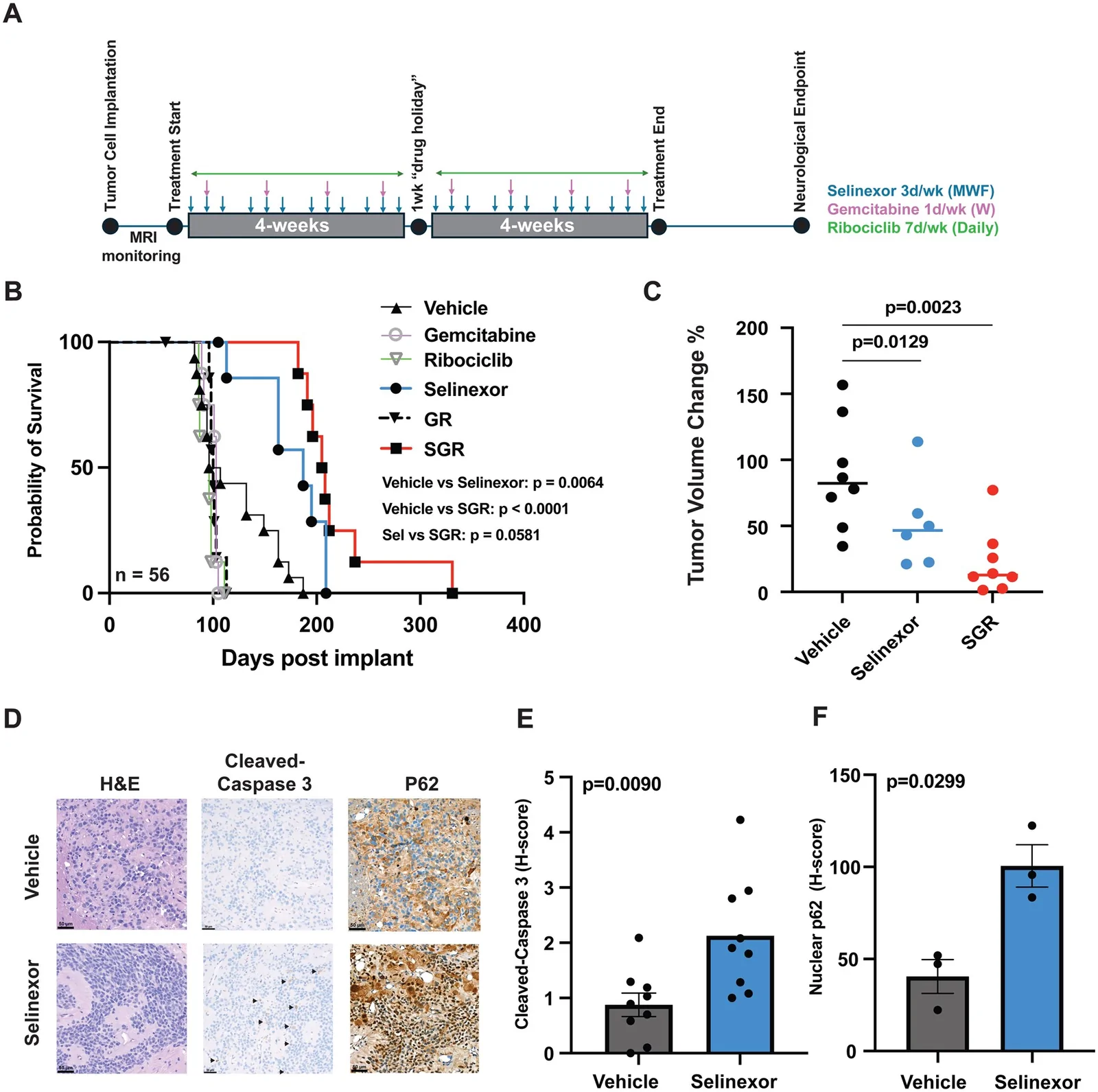
(A) Representation of the study design for preclinical study. (B) Survival of NSG mice allografted with ZR-driven EPN treated with either vehicle or drugs for 8 weeks (*n* = 8/arm). (C) Change in tumor volumes during treatment period in NSG mice with ZR-driven EPN treated with either vehicle (*n*=16) or drugs (*n* = 8/arm). (D) Representative images of ZR-driven EPN tumors treated with Saline or 10 mg/kg selinexor stained with antibodies for cleaved-caspase-3 & p62. (E) and (F) H-score of nuclear cleaved-caspase and p62 levels in ZR-EPN mice (*n* = 3) treated with vehicle or 10 mg/kg selinexor for 4 weeks.

## Discussion

Ependymomas are relentless malignant pediatric CNS tumors in children, and patients are in desperate need of novel therapies.^33^ The majority of supratentorial ependymomas are driven by ZFTA gene fusions, most commonly ZR.^11–13^^,^^16^ Adult supratentorial EPNs are also primarily characterized by ZR gene fusions. Despite extensive evidence that the ZR fusion is sufficient to drive tumor formation, efforts to directly target this protein have been hampered by the relatively disordered structure of the ZR protein and the inability to design effective small molecules. As an alternative approach, using biochemical assays, we provide evidence that ZFTA alters the protein-protein interactome, with a shift toward association with high buoyant density complexes upon gene fusion. Through immunoprecipitation and mass spectrometry of ZR in mouse models, we mapped proteins that interact with ZR, such as known members of the NF-ĸB complex and many novel interacting proteins including Npm1, Cdk1, Smarca44, Mcm6, and Xpo1. A focused CRISPR/Cas9 dependency screen provided evidence that many of these interaction partners are required for tumor cell survival, such as Xpo1, Carm1, and Hspa5. This provides a framework for therapeutic discovery that could be readily applied to study other oncoproteins, particularly those where cellular models are challenging to grow, such as pediatric and adult brain tumors. A caveat from this approach is that weaker interacting proteins, such as those existing within biomolecular condensates are likely to be missed by more stringent IP-mass spectrometry methods.^15^ Future efforts to screen ZR-interacting proteins using technologies such as proximity labeling could unravel additional lead targets.

To further demonstrate the ability of our framework to identify lead therapeutic targets, we focused on a “hit” *Xpo1*, which showed preferential sensitivity in tumor cells versus normal cell counterparts. Surprisingly, we found that XPO1 regulates ZR localization and that specific levels of ZR expression in the nucleus are needed for continued cell proliferation. In a similar, yet mechanistically distinct fashion, EWS-FLI1 expression in Ewing’s Sarcoma is fine-tuned by tripartite motif-containing 8 (TRIM8), an E3 ubiquitin ligase that ubiquitinates and targets EWS-FLI1 for degradation to fine-tune its expression.^34^ We reasoned that XPO1 could be serving a similar role by titrating appropriate levels of ZR nuclear expression to maintain oncogenic expression while safeguarding against oncogene-induced cell cycle arrest. A broader idea that emerges is the idea of hyperactivation of oncoprotein activity, which could represent a tumor vulnerability, reminiscent of concepts of oncogene-induced senescence.

To this date, our study and many others have focused almost entirely on the nuclear role of ZR as an oncogenic transcription factor.^11^^,^^13–16^ Our study demonstrates that ZR exists within cellular complexes, also outside the nucleus, where we have mapped novel interacting proteins. An entirely open area of investigation is the potential role of ZR in engaging with non-nuclear proteins, and how these interactions mediate oncogenicity and represent therapeutic targets.

One of the challenges of translating molecular findings in EPN has been the relative scarcity of PDX models compared to other brain tumors, such as high-grade glioma.^33^ More recently, cell lines of ZR-EPN have become available; these either do not grow *in vivo* or take more than approximately 8-12 months to develop tumors, making *in vivo* preclinical studies in many models very challenging to perform consistently and rigorously. As an example, combination therapy in 1425-NS, our “fastest” and most consistent model, required nearly a year to complete a preclinical trial. These challenges are compunded by a low incidence of these tumors and a modest sucess rates of implantation into mice to generate a disease model . This is likely a major reason why efforts to translate molecular findings to patients in the past have progressed slowly, and EPN continues to be treated with decades-old therapy.

Despite these limitations, our study pinpoints a lead target in ZR ependymoma. We demonstrated that a panel of ZR-driven pediatric models is responsive to selinexor treatment at doses capable of crossing the blood-brain barrier (BBB). We extend these findings *in vivo* and show that selinexor monotherapy and combination therapy with GR can slow tumor growth and significantly extend survival in an orthotopic xenograft tumor-bearing mice. While monotherapies are not likely to be curative against relapsed EPN, we would argue that these patients currently have limited options outside of re-irradiation, and there are very limited clinical trials available.^1^ SGR combination therapy should be strongly considered for clinical trial evaluation in both pediatric and adult EPN patients.

## Supplementary Material

noag100_Supplementary_Data

## Data Availability

Data will be made available through the St Jude Cloud, and also directly available upon request to the corresponding authors.
