# Supplementary material for: Nuclear export as a therapeutic vulnerability in ZFTA-RELA ependymoma
Source: Neuro Oncol. 2026 May 3;28(10):2472–84. doi: 10.1093/neuonc/noag100 (PMC13498431; doi:10.1093/neuonc/noag100)
Supplement: Supplementary file 1 [file noag100_supplementary_data.zip › Supplementary and Main Figure Legends..docx]

**Supplementary Figure 1** (A) Coomassie gel stain of HA immunoprecipitated cell lysates collected from HA-ZR IUE derived cells vs RELA KO controls (n = 3). (B) Mass spectrometry analysis of ZR IUE cells vs RELA KO controls showing iBAQ values of ZR (C) GO ontology analysis of ZR interacting proteins detected by mass spectrometry as compared to RELA KO controls. (D) CRISP screen results for radial glia cells, putative cell of origin for ependymoma. (E) Percentage of Indel reads in RGC cell line electroporated with either sg(control) or sg(XPO1) guides. 0bp indicates reads of full length gene‘Out of frame’ and ‘Inframe’ indicates percent of knockdown

**Supplementary Figure 2.** (A) read count comparison for ZFTA-RELA downstream target genes for Radial glia cells or Radial glia cells expressig ZFTA-RELA treated with DMSO or 1µM Selinexor for 48 hours. (B) GO onotology analysis of ZR IUE cells (M0726) describing significantly up-regulated or down-regulated pathways in cells treated with vehicle or selinexor. (C) Biological replicate of experiment depicted in Fig3D. (D) additional patient derived xenogeraft models treated with increasing concentration of Selinexor

**Supplementary Figure 3.** (A) Change in tumor volume in mice with ZR driven EPN treated with Vehicle or GR (n=8/arm). (B) Tumor volumes in individual mice with ZR driven EPN treated with Vehicle or drugs until end point. (C) H-score of Cytosolic RELA levels in ZR-EPN mice (n=3) treated with Vehicle or 10mg/kg Selinexor. (D) H-score of cytosolic p62 levels in ZR-EPN mice (n=3) treated with Vehicle or 10mg/kg Selinexor. (E) Body weights of ZR-EPN mice treated with Vehicle or Selinexor (10mg/kg or 20 mg/kg), n=8/arm
